# *Morc4* is a novel functional gene associated with lipid metabolism in BXD recombinant inbred population

**DOI:** 10.3389/fcvm.2025.1570729

**Published:** 2025-06-18

**Authors:** Zhanyi Yang, Jiaai Xu, Xiaoyu Yang, Pengcheng Yi, Jian Ruan, Yingying Wu, Yushan Li, Geng Tian, Fuyi Xu, Jia Mi, He Li, Chunhua Yang

**Affiliations:** ^1^ Shandong Technology Innovation Center of Molecular Targeting and Intelligent Diagnosis and Treatment, Binzhou Medical University, Yantai, Shandong, China; ^2^ Pharmacology and Toxicology Department, Yantai Food and Drug Inspection Center, Yantai, Shandong, China; ^3^ Department of Stomach and Intestine, Yantai Affiliated Hospital of Binzhou Medical University, Yantai, Shandong, China

**Keywords:** BXD mice, lipid metabolism, *MORC4*, TC, TG

## Abstract

**Aim:**

The dysregulation of hepatic lipid metabolism is closely associated with dyslipidemia. Previous research suggested that Hepatic *Morc4* may play a role in regulating lipid metabolism. This research aims to elucidate the function of MORC4 in hepatic lipid metabolism, thereby improving the understanding of the molecular mechanisms underlying lipid metabolism disorders.

**Methods:**

Data regarding circulating lipid traits and hepatic *Morc4* expression in BXD mice were obtained from GeneNetwork. An Expression-Based Phenome-wide Association Study (ePheWAS), correlation analysis, and gene enrichment analysis were conducted to explore the relationship between *Morc4* expression and hepatic lipid metabolism. *in vitro*, the levels of total cholesterol (TC) and triglycerides (TG), lipid accumulation, and the expression of lipid metabolism-related genes were assessed subsequent to *MORC4* knockdown/overexpression in hepatocytes. *in vivo*, the impact of *Morc4* knockout on lipid metabolism-related traits in mice was examined using the IMPC database.

**Results:**

Hepatic *Morc4* level was found to be negatively correlated with plasma free fatty acids and triglycerides in BXD mice. Further analysis indicated that genes associated with *Morc4* were enriched in the cholesterol metabolic pathway. In hepatocytes, *MORC4* knockdown significantly elevated total TC/TG levels, as well as enhanced lipid accumulation. Whereas *MORC4* overexpression restored total TC/TG levels, along with lipid accumulation in knockdown cell lines. Furthermore, *MORC4* knockdown led to an increased expression of genes associated with cholesterol synthesis (*HMGCR*), varying levels of genes implicated in the uptake of fatty acids and cholesterol (*PCSK9, PLTP, CD36*), and a decrease in the levels of genes involved in triglyceride hydrolysis (*APOC2, APOA4, LIPG, LIPA*). *MORC4* overexpression reversed the observed alterations in the expression levels of these genes. According on the IMPC database, *Morc4* knockout in mice resulted in increased fat mass, fat/body weight ratio, and elevated cholesterol level and ratio.

**Conclusion:**

This study identifies *MORC4* as a crucial regulator of hepatic lipid metabolism and underscores its potential as a therapeutic target for disorders related to lipid.

## 1 Introduction

The liver is the principal site for maintaining lipid and cholesterol homeostasis, facilitating processes such as lipid oxidation, packaging, and secretion of excess lipids to peripheral tissues, while also serving as a key provider of lipid substrates for the body (1–3). Disruptions in hepatic lipid metabolism can contribute to the development of various metabolic syndrome including hyperlipidemia. Importantly, hepatic lipid metabolism is influenced by a complex interplay of environmental factors and genetic predispositions (4–6).

The BXD recombinant inbred strains derived from the interbreeding of C57BL/6J and DBA/2J mice is a significant genetic reference population (7, 8). Compared to single-generation genetic mapping, the BXD strains demonstrate an approximate fourfold increase in recombination frequency, thereby improving the resolution of genetic mapping (9, 10). Furthermore, BXD mice exhibit considerable variability in lipid metabolism, rendering them an optimal genetic model for the exploration of lipid metabolic processes (11, 12). These mice are extensively employed in the analysis of genetic variations linked to the etiology of human diseases, highlighting significant differences in lipid metabolism and thus providing a foundational genetic framework for the investigation of lipid metabolic mechanisms.

In our prior research, Morc4 was identified as a pivotal hub gene significantly linked to lipid metabolism in BXD mice (13). MORC4 is a highly conserved member of the MORC family, which comprises four members (MORC1-4) that exhibit a shared an N-terminal ATPase-like ATP-binding domain and a CW-type cysteine-rich zinc finger domain (14). As nucleolar-associated nuclear proteins, MORC members are considered important transcriptional regulators involved in various biological processes. In the prior research, the differentially expressed genes in livers from normal-fed mice and high-fat diet-fed mice were analyzed and enriched in various biological processes. A gene module, which includes *Cd36* and *Mogat1*, was enriched in lipid metabolic processes. CD36 functions as a critical sensor of fatty acids and serves as a regulatory element in lipid metabolism (15). MOGAT1 is a lipogenic enzyme involved in the regulation of the lipolytic process (16). Evidence supports the identification of CD36 as a pharmacological target for the modulation of fatty acid uptake (17). Additionally, compounds that target MOGAT1 may represent promising therapeutic options for the prevention of hepatic steatosis (18). *Morc4* was identified as a pivotal hub gene of this gene module. A recent proteome-Wide Mendelian Randomization analysis identifies *MORC4* as a genetic risk locus associated with acute pancreatitis (19). These suggest MOCR4 may play a critical role in lipid metabolism. However, the mechanism by which MOCR4 regulates lipid metabolism remains unclear.

This study aimed to elucidate the role of MORC4 in regulating lipid metabolism through the integration of systems genetics analysis in BXD mice and subsequent *in vitro* experimental validation. The results revealed that *MORC4* knockdown led to elevated levels of hepatocytic triglycerides and cholesterol, primarily through the modulation of gene expression associated with cholesterol metabolic pathways. These findings establish a significant relationship between MORC4 and lipid metabolism in the liver, suggesting potential new therapeutic target for metabolic liver disorders.

## 2 Materials and methods

### 2.1 BXD liver transcriptomic data Set

The BXD liver transcriptome dataset was sourced from GeneNetwork website (https://gn1.genenetwork.org/webqtl/main.py) (20). The parameters were set as follows: Species, Mouse (mm10); Group, BXD Family; Type, Liver mRNA; Data Set, EPFL/LISP BXD CD Liver Affy Mouse Gene 1.0 ST (Aug18) RMA; Get Any, Morc4. The transcriptome dataset with ID10606948 was obtained. GraphPad Prism 8.0 was used for data visualization to display the changes in Morc4 gene expression levels in BXD mice.

### 2.2 Expression-based phenome-wide association study (ePheWAS)

The ePheWAS data were obtained from the GeneNetwork system genetics platform (https://systems-genetics.org/ephewas) (21). The data were downloaded from this website for the experiment [Tissues: GN432, EPFL/LISP BXD CD Liver Affy Mouse Gene 1.0 ST (Apr13) RMA, Gene: Morc4], which included Free Fatty Acids (FFA) in plasma (ID: 17813) and Triglycerides in plasma (ID: 17809). The data were ultimately visualized using GraphPad Prism 8.0.

### 2.3 Gene correlation analysis

The Top 2,000 genes associated with Morc4 in the BXD mouse liver were obtained from GeneNetwork (in conjunction with BXD liver transcriptome data). Subsequently, gene correlation analysis was performed under the “Compute Correlation” option to identify the Top 2,000 genes associated with *Morc4*.

### 2.4 Gene function enrichment analysis

Gene function enrichment analysis of Top 2,000 genes was conducted utilizing the hypergeometric test provided by the WebGestalt platform (https://www.webgestalt.org/) (22). The objective of this analysis was to identify biological terms that are significantly overrepresented, including KEGG pathways and Gene Ontology (GO) categories.

### 2.5 International mouse phenotyping

The lipid metabolism-related phenotype data in *Morc4* knockout mice were obtained from the International Mouse Phenotyping Consortium (IMPC) (https://www.mousephenotype.org/). IMPC employs high-throughput workflows to phenotype gene knockout mouse strains. For each gene, a homologous gene knockout mouse strain is generated, consisting of seven males and seven females. Wild-type (WT) control mice are analyzed in batches over an extended time period (23). The data obtained from IMPC, which have been analyzed using the PhenStat R package developed by IMPC (24), were ultimately visualized using GraphPad Prism 8.0.

### 2.6 Cell lines and cell culture

In this research, THLE-2 cells, which are immortalized adult liver epithelial cells, and HepG2 cells, which represent human hepatocellular carcinoma, serve as models for investigating lipid metabolism. HepG2 and THLE cells were purchased from Wuhan Pricella Biotechnology Co., Ltd. The cells were cultured under standard conditions using two types of media: RPMI 1640 for THLE-2 (Qidu, China) and DMEM for HepG2 (Qidu, China). The media were supplemented with 10% fetal bovine serum (FBS) (Vazyme, China) and 10% penicillin-streptomycin (Solarbio, China). Cells were maintained in a humidified incubator at 37°C with 5% CO₂. To induce lipid dysregulation, a 0.5 mmol/L oleic acid (OA) solution (Sangon Biotech, China) was used to incubate the cells for 24 h.

### 2.7 MORC4 knockdown and overexpression

Two small interfering RNAs (siRNAs) designed for the knockdown of *Morc4* were synthesized by Tsingke Biotechnology Co., Ltd. (China). The specific sequences of the siRNAs are provided in Table 1. The Flag-MORC4 plasmid, utilized for the overexpression of *Morc4*, was obtained from Hunan Fenghui Biotechnology Co., Ltd. (China). In knockdown and overexpression experiments, siRNA and Flag-MOCR4 were transfected into cells separately for a duration of 48 h, followed by treatment with 0.5 mmol/L oleic acid (OA) for 24 h. For the rescue experiments, cells were first transfected with siRNA for 24 h. Subsequently, Flag- MOCR4 was transfected into the cells for another 24 h, followed by OA treatment for an additional 24 h. The control groups, which did not receive OA treatment, adhered to the same protocols, with the exception of the OA exposure.

**Table 1:** siRNA used for RNA interference.

Name	Sequence
siMORC4-1	5′-GAUCGGGUAUGACUCAGAA 3′-UUCUGAGUCAUACCCGAUC
siMORC4-2	5′-CAAUACCGAAGGUUCCUGA 3′-UCAGGAACCUUCGGUAUUG

### 2.8 Quantitative real-time PCR (qRT-PCR)

THLE-2 and HepG2 cells were washed with PBS. Total RNA was extracted using Trizol reagent, following the manufacturer's protocol (Vazyme, China). Reverse transcription was conducted utilizing the HiScript III cDNA Synthesis Kit (Vazyme, China), following the protocol to remove genomic DNA contamination. qRT-PCR was conducted on the Applied Biosystems (Thermo Fisher, USA) as follows: initial denaturation at 95°C for 10 min, 36 cycles of 95°C for 25 s, 60°C for 1 min, and 72°C for 30 s. The mRNA expression levels were quantified employing 2−*ΔΔ*Ct method, with β-actin serving as the internal control. The results were presented as relative expression changes in comparison to the control. The primers were synthesized by Tsingke Biotechnology Co. (China), Ltd., and their sequences are detailed in Table 2.

**Table 2:** Primers used for real-time PCR.

Gene	Primer sequence
*Morc4*	5′-CATCGCGGAGCTGCTAGATAA 3′-TCCATCATCGGTAAAGGTCAAAC
*Hmgcr*	5′-TGATTGACCTTTCCAGAGCAAG 3′-CTAAAATTGCCATTCCACGAGC
*Lipa*	5′-TCTGGACCCTGCATTCTGAG 3′-CACTAGGGAATCCCCAGTAAGAG
*Lipg*	5′-GATGGACGATGAGCGGTATCT 3′-CGCATCCGTGTAAAGCTGG
*Apoc2*	5′-TGTCCTCCTGGTATTGGGATTT 3′-TGTCTTCTCGTACAGGTTCTGG
*Pltp*	5′-AAGAGCGGATGGTGTATGTGG 3′-ATGGGGAGTCAATCACTGCTG
*Pcsk9*	5′-ATGGTCACCGACTTCGAGAAT 3′-GTGCCATGACTGTCACACTTG
*Cd36*	5′-AAGCCAGGTATTGCAGTTCTTT 3′-GCATTTGCTGATGTCTAGCACA
*Apoa4*	5′-CCCAGCAACTCAATGCCCT 3′-CCTTCAGTTTCTCCGAGTCCT
*β*-*Actin*	5′-CCCAGCAACTCAATGCCCT 3′-CCTTCAGTTTCTCCGAGTCCT

### 2.9 Total cholesterol and triglyceride assays

After 24 h of siRNA transfection in THLE-2 and HepG2 cells, cultures were washed with PBS and detached utilizing 0.25% trypsin-EDTA (Gibco, USA). Cells were lysed for 30 min in WB-IP lysis buffer (Beyotime, China) to obtain homogenates. Protein concentrations were determined using a Bicinchoninic Acid (BCA) Protein Assay Kit (Beyotime, China) to ensure uniformity across samples. The levels of total cholesterol (TC) and triglycerides (TG) were assessed employing assay kits from Nanjing Jiancheng Bioengineering Institute (China), according to the manufacturer's instructions. Absorbance readings at 500 nm was measured using a BioTek microplate reader (BioTek, USA), and lipid concentrations were calculated based on standard curves to ensure accurate data assessment.

### 2.10 Oil red O staining

THLE-2 and HepG2 cells were fixed with 4% paraformaldehyde for 30 min to maintain cellular morphology. Following fixation, the cells were dehydrated through treatment with 60% isopropanol for 10 min to facilitate optimal dye uptake. Subsequently, the cells were stained with Oil Red O working solution (Meilunbio, China) for an hour to visualize lipid content effectively. After staining, excess dye was removed by washing the cells three times with phosphate-buffered saline (PBS) (Qidu, China), ensuring clear visualization of lipid droplets. The stained cells were then examined and documented using a Zeiss microscope (Germany), allowing for a detailed assessment of lipid accumulation within the cells.

### 2.11 Statistical analyses

The experimental data are presented as mean ± standard deviation (SD). Statistical analysis was performed using GraphPad Prism 8.0. Statistical evaluations between two groups were carried out using Student's t test (Un-paired, two-tailed), and for comparison between multiple groups, one-way ANOVA was used. Pearson correlation analysis was employed for comparisons involving correlations. Statistical significance was set at *p < 0.05, **p < 0.01, and ***p < 0.001.

## 3 Results

### 3.1 Significant correlation between *Morc4* expression and lipid metabolism in BXD mice

**Figure 1:**
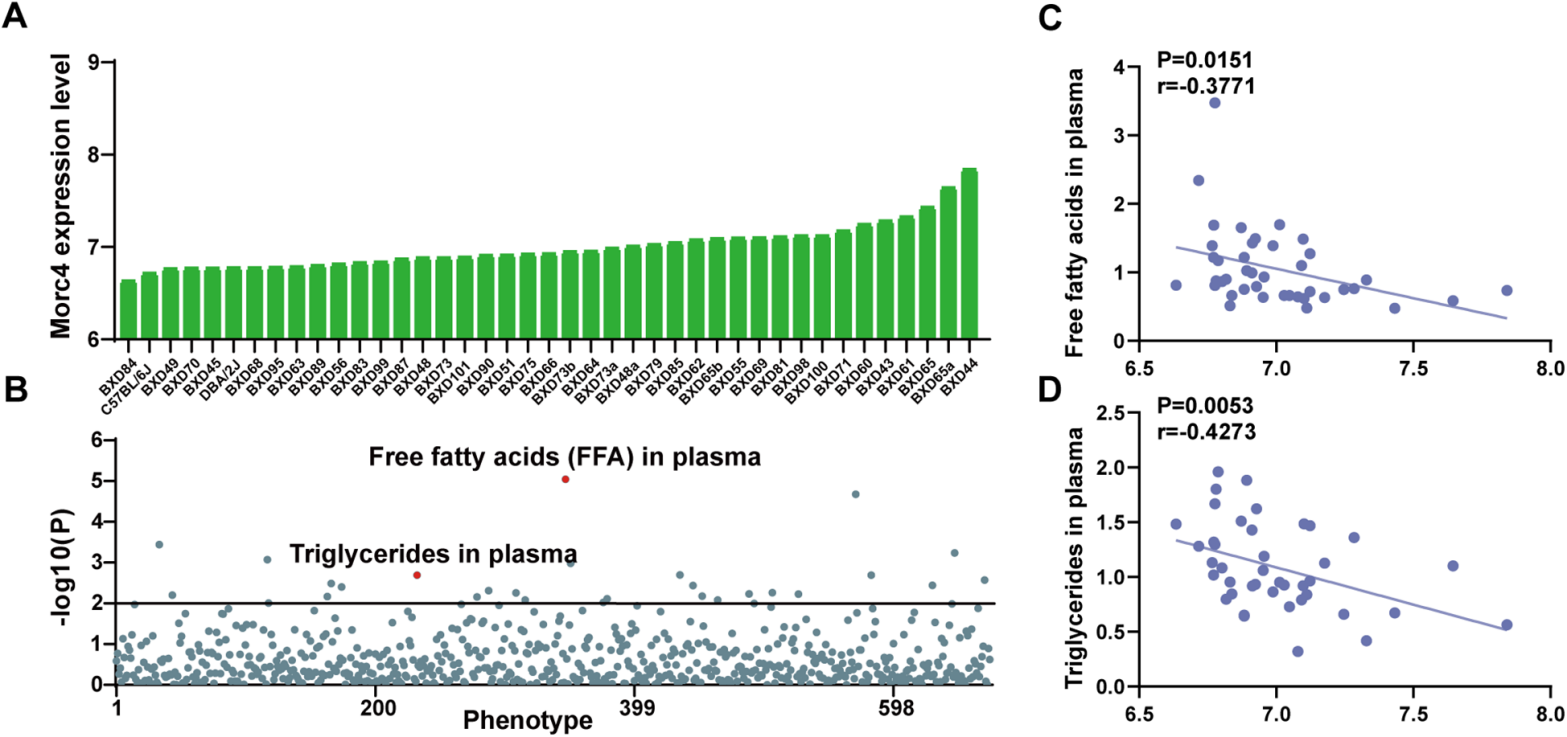
The expression levels of the hepatic *Morc4* associated with hepatic lipid metabolism phenotypes. **(A)** hepatic *Morc4* expression levels across 41 BXD mouse strains. **(B)** Manhattan plots from ePWAS emphasizing the metabolic phenotypes associated with *Morc4* expression in BXD mice. Each point represents a distinct metabolic phenotype, with the red horizontal line denoting the threshold for statistical significance (log10 *p* > 2). **(C,D)** Scatterplots depicting the correlation between *Morc4* expression and plasma lipid metabolism levels in the liver of BXD mice. Each point corresponds to an individual BXD strain. Pearson correlation coefficients were calculated to evaluate the relationship.

The expression levels of Morc4 in the liver from 41 BXD mouse strains were assessed. An average expression level of 6.99 ± 0.20 SD was observed with the lowest expression at 6.635 in BXD84 and the highest at 7.841 in BXD44 (Figure 1A). Further ePheWAS of *Morc4* in BXD mice delineated associations between hepatic *Morc4* expression and lipid metabolism-related phenotypes (Figure 1B). These phenotypes encompass plasma free fatty acids [ID: 17813, -log10(p) = 5.05] and plasma triglycerides [ID: 17809, -log10(p) = 2.69]. Furthermore, a negative correlation was observed between *Morc4* expression and levels of plasma free fatty acids (ID: 17811, *P* = 0.0151, *r* = 0.3771 Figure 1C) and plasma triglycerides (ID: 17809, *P* = 0.0053, *r* = 0.4273 Figure 1D).

### 3.2 MORC4 affects hepatic lipid metabolism *in vitro*

**Figure 2:**
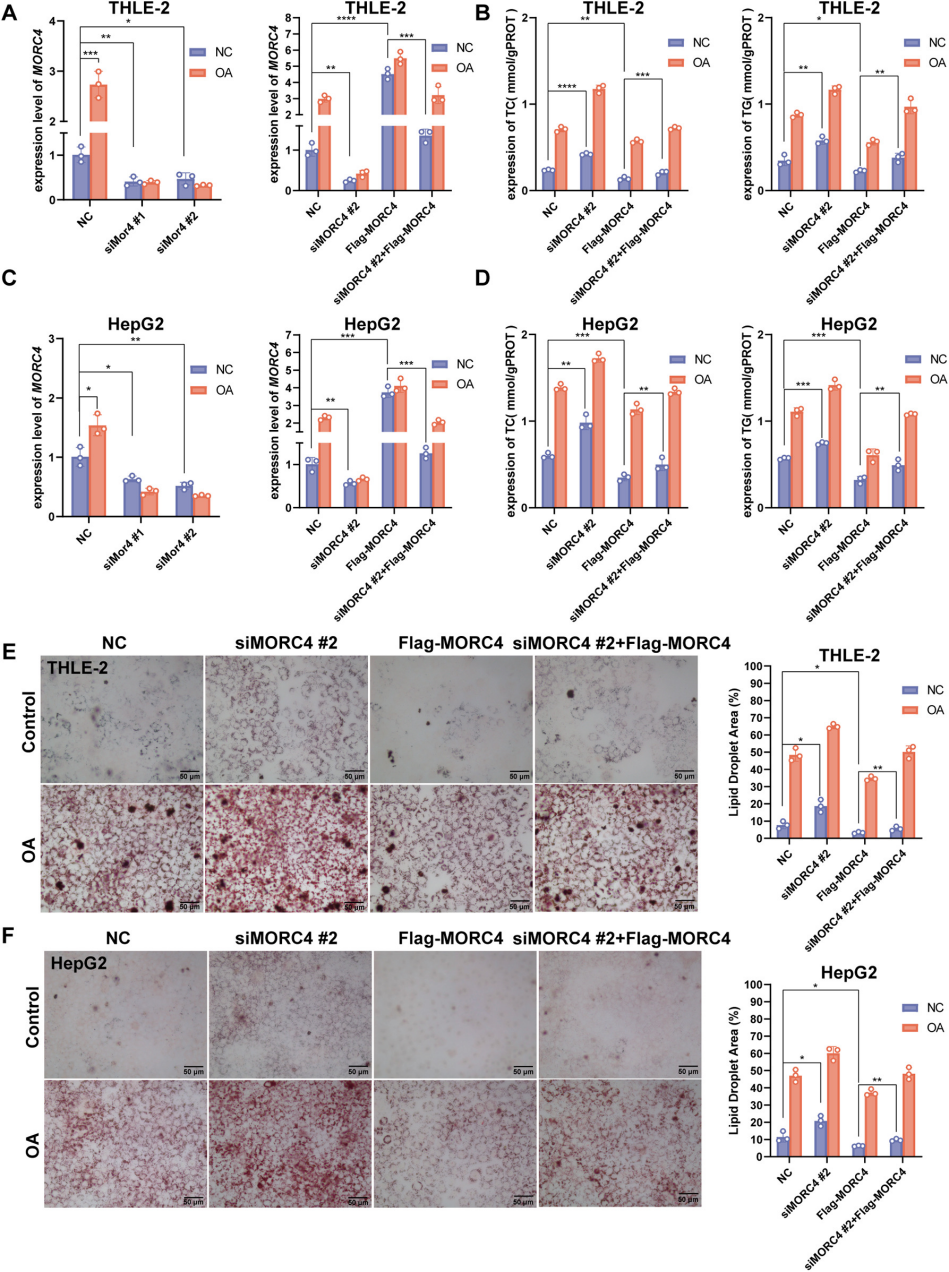
The role of MORC4 on lipid metabolism in THLE-2 and HepG2 cells. The knockdown and overexpression of *MORC4* in THLE-2 cells **(A)** and HepG2 cells **(C)** were analyzed using qPCR experiment. The total cholesterol (TC) and triglyceride (TG) levels following *MOCR4* knockdown and overexpression in THLE-2 cells **(B)** and HepG2 cells **(D)** were measured by assay kits. The accumulation of lipid droplets in THLE-2 cells **(E)** and HepG2 cells **(F)** after MOCR4 knockdown and overexpression were detected with Oil Red O staining. OA, oleic acid; *P < 0.05, **P < 0.01, ***P < 0.001; *n* = 3.

To further elucidate the role of MORC4 in hepatic lipid metabolism, MORC4 expression was knocked down in the THLE-2 and HepG2 cell lines using two different siRNAs (siMORC4#1 and siMORC4#2) and siMORC4#2 presents better knockdown efficiency. Moreover, the knockdown of MORC4 expression were rescued by the transfection of Flag-MORC4 (Figures 2A,B; Supplementary Figure S1). THLE-2 and HepG2 cells were exposed to oleic acid (OA) to induce lipid metabolism dysregulation (25, 26). The total cholesterol (TC) and triglyceride (TG) levels within the cells were quantified using assay kits. Notably, MORC4 knockdown led to a significant increase in TC and TG levels in both normal and OA-treated cells, while MORC4 overexpression significantly downregulated TC and TG levels in both normal and OA-treated cells. Additionally, MORC4 overexpression in siRNA-transfected cell lines restored the TC and TG levels Figures 2B,D). Similarly, Oil Red O staining revealed that MORC4 knockdown exacerbated the accumulation of lipid (droplets in both normal and OA-treated cells, which could be mitigated by the overexpression of MORC4 (Figures 2E–H). Furthermore, a marked elevation of MORC4 level was observed in THLE-2 and HepG2 cells following OA treatment (Figures 2A,C; Supplementary Figure S1). These results indicate that MORC4 plays a critical role in the accumulation of cholesterol, triglycerides, and lipid droplets, thereby highlighting its significant involvement in hepatic lipid metabolism.

### 3.3 Genes associated with *Morc4* involved in lipid metabolism functions and pathways

**Figure 3:**
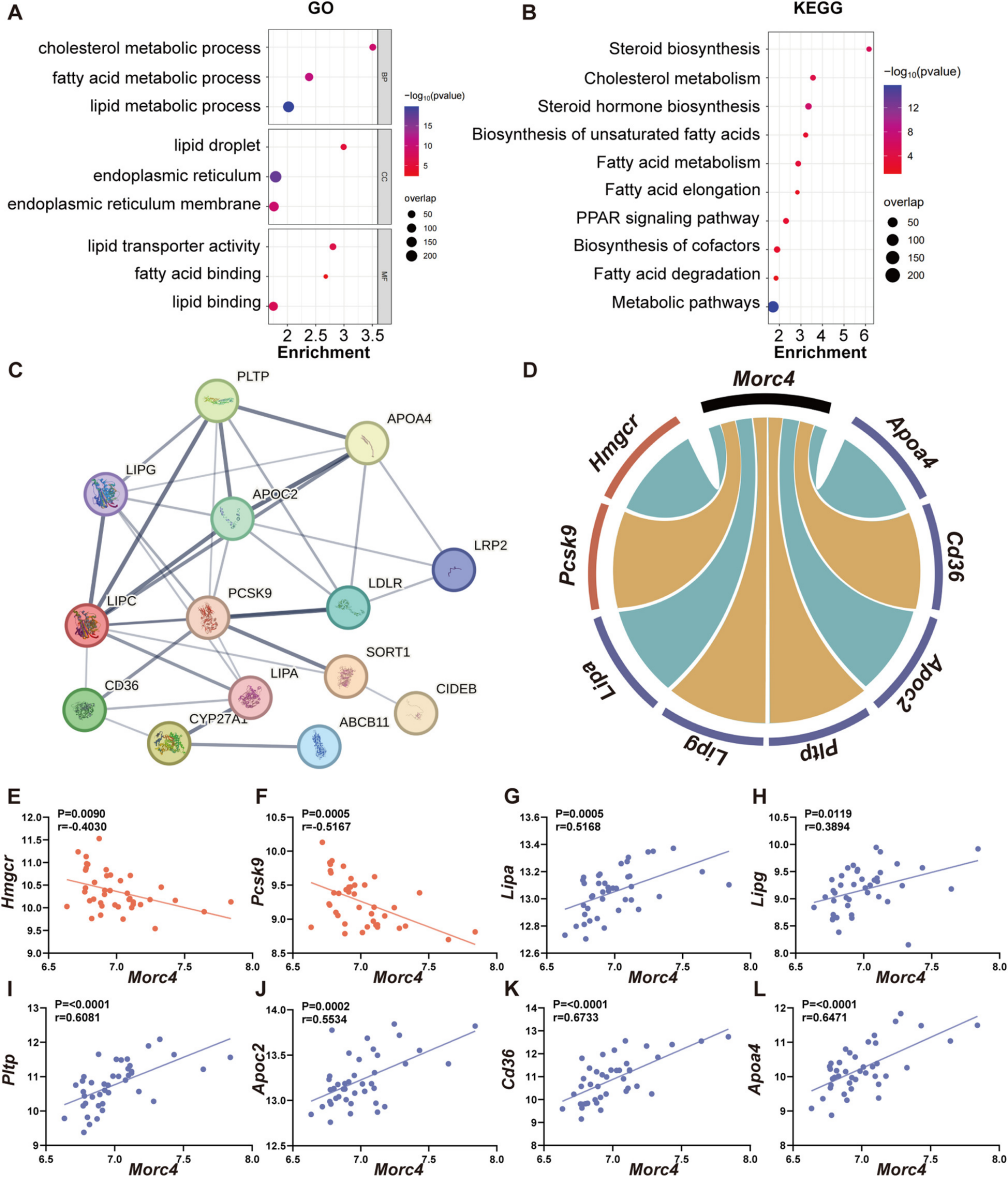
The relationship between Morc4 and cholesterol metabolism pathways. The GO enrichment bubble plot **(A)** and KEGG enrichment bubble plot **(B)** of the top 2,000 genes related to Morc4.The *x*-axis represents the enrichment ratio, the *y*-axis lists the GO terms, the bubble size reflects the gene count, and the color indicates the FDR value. **(C)** The protein-protein interaction (PPI) network related to the cholesterol metabolism pathway highlights genes with an interaction score greater than 0.4 (indicating moderate confidence), **(D)** sourced from the STRING database (https://www.string-db.org/). In the cholesterol metabolism pathway, Morc4 shows significant correlations with eight key lipid metabolism genes, with negative correlations shown in blue and positive correlations shown in red. **(E–L)** A scatter plot of the correlation between these eight genes and MORC4 is provided, along with the correlation coefficient (r) and corresponding *p*-values for each plot.

In order to elucidate the role of MORC4 in the regulation of hepatic lipid metabolism, Pearson correlation analysis of *Morc4* against the other transcripts using the BXD mice liver transcriptome was employed on GeneNetwork. The top 2,000 genes exhibiting strong correlation with *Morc4* were identified (*r* > 0.3, *P* < 0.05) and analyzed with gene enrichment analyses. GO analysis demonstrated significant enrichment in biological processes (BP) associated with cholesterol metabolism (*P* < 0.0001) and fatty acid metabolism (*P* < 0.0001), cellular components (CC) linked to lipid droplets (*P* < 0.0001) and the endoplasmic reticulum (*P* < 0.0001), and molecular functions (MF) related to lipid transporter activity (*P* < 0.0001) and fatty acid binding (*P* = 0.0036) (Figure 3A). Pathway enrichment analysis was performed by KEGG analysis which highlighted enrichment in pathways associated with metabolic pathways (*P* < 0.0001), (Figure 3B). Particularly, 14 genes were significantly enriched in the cholesterol metabolism pathway (*P* < 0.05), (Supplementary Table S1). The protein-protein interaction network for these 14 genes is illustrated in Figure 3C. Among these, seven key genes were identified as being closely related to cholesterol and fatty acid endocytosis, as well as triglyceride degradation. Six of these genes demonstrated significant positive correlations with *Morc4* expression, including *Lipa* (*r* = 0.5168, *P* = 0.0005), *Lipg* (*r* = 0.3189, *P* = 0.0119), *Pltp* (*r* = 0.6081, *P* < 0.0001), *Apoc2* (*r* = 0.5534, *P* = 0.0002), *Cd36* (*r* = 0.6733, *P* < 0.0001), and *Apoa4* (*r* = 0.6471, *P* < 0.0001), (Figures 3D,G–L). In contrast, *Pcsk9* exhibited a significant negative correlation with *Morc4* expression (*r* = −0.5167, *P* = 0.0005), (Figures 3D,F). Furthermore, a strong negative correlation was observed between *Morc4* and *Hmgcr* (*r* = −0.4030, *P* = 0.0090), (Figures 3D,E),which is recognized as a critical rate-limiting enzyme in cholesterol biosynthesis (27).

### 3.4 MORC4 influences the expression of genes associated with lipid metabolism *in vitro*

**Figure 4:**
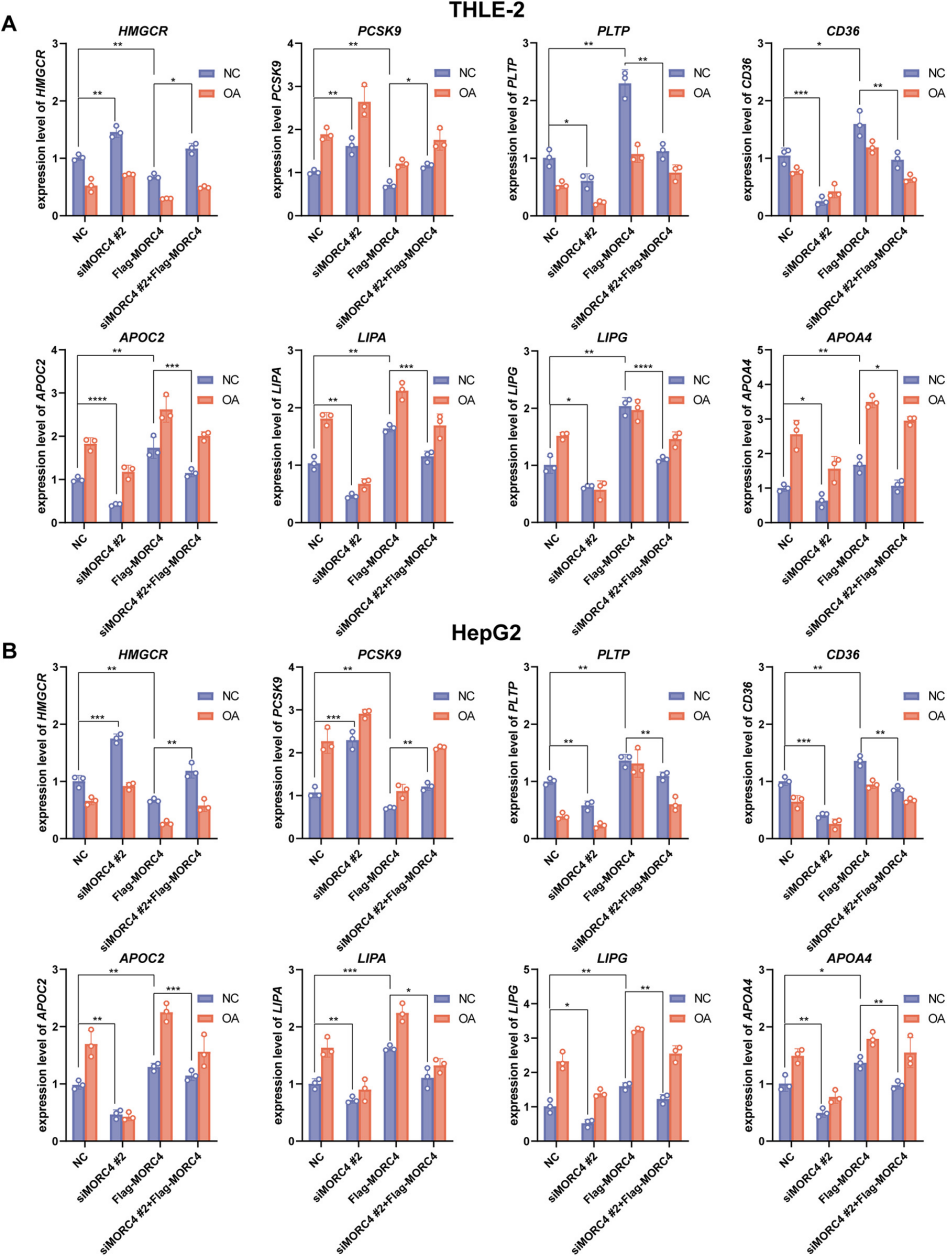
The role of MORC4 in regulating the expression levels of lipid metabolism-related genes. After changes in MORC4 expression levels, alterations in the expression of lipid metabolism-related genes in THLE-2 cells **(A)** and HepG2 cells **(B)** were observed. *P < 0.05, **P < 0.01, ***P < 0.001; *n* = 3.

To assess the interaction between *MORC4* and eight identified genes, the expression levels of the identified genes in normal and OA-treated THLE-2 and HepG2 cell lines were quantified by qPCR analysis following MORC4 knockdown and overexpression. Consistent with the results of Pearson correlation analysis, MORC4 knockdown led to a decrease in the expression of *LIPA*, *LIPG*, *PLTP*, *APOC2*, *CD36* and *APOA4,* while simultaneously increasing the expression of *HMGCR* and *PCSK9.* However, MORC4 overexpression rescued the alteration in these genes’ expression levels caused by MORC4 knockdown (Figure 4). Moreover, OA treatment significantly increased the expression of *APOC2*, *LIPA*, *LIPG*, and *APOA4*, similar to the effects of *MORC4*. These results suggest that *MORC4* may regulate lipid metabolism by modulating the expression of genes involved in cholesterol metabolism and biosynthetic pathways.

### 3.5 Impact of *Morc4* knockout on lipid metabolism-related phenotypes in mice

**Figure 5:**
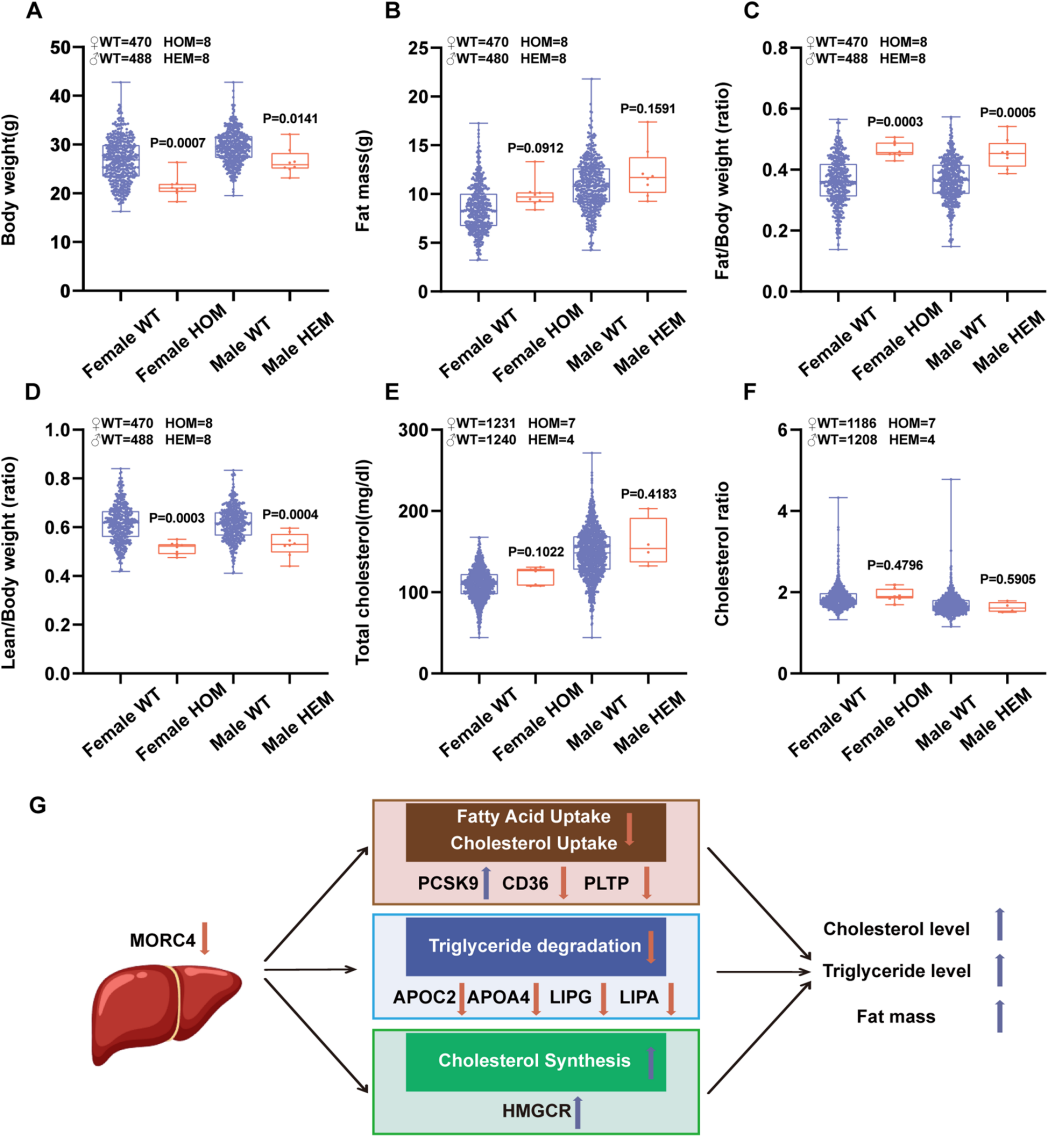
Alterations in lipid metabolism phenotypes following *Morc4* knockout in IMPC database. The residuals of whole-body lipid metabolism-related traits were adjusted for genotype and sex in mice that are heterozygous for a *Morc4* gene-trap allele. **(A)** Body weight, **(B)** Fat mass, **(C)** Fat/body weight ratio, **(D)** Lean/body weight ratio, **(E)** Total cholesterol, **(F)** Cholesterol ratio, **(G)** Proposed model illustrating that MORC4 regulates hepatic lipid metabolism. The downregulation of *Morc4* expression leads to reduced uptake of free fatty acids/cholesterol, as well as a decrease in triglyceride hydrolysis, while concurrently enhancing cholesterol synthesis. This interplay contributes to the dysfunction of lipid metabolism.

The association between *Morc4* and lipid metabolism-related phenotypes in mice was examined utilizing the International Mouse Phenotyping Consortium (IMPC) database. Compared to wild-type mice, *Morc4* knockout mice demonstrated a reduction in body weight (Figure 5A), an increase in the fat mass and fat/body weight ratio (Figures 5B,C), a decrease in the lean/body weight ratio (Figure 5D), as well as elevated total cholesterol levels (Figure 5E) and cholesterol ratios (Figure 5F). In summary, these results suggest that the silence of *Morc4* disrupts cholesterol metabolism and contributes to abnormal weight distribution in mice.

## 4 Discussion

The liver serves as a central organ for the synthesis of fatty acids and the circulation of lipids, thereby playing a crucial role in the regulation of lipid metabolism homeostasis. Within the liver, fatty acids (FAs) derived from various sources are utilized for the synthesis of triglycerides, which may subsequently undergo oxidation, storage, or secretion into the systemic circulation. Disruptions in these metabolic processes can lead to the accumulation of intrahepatic triglycerides, potentially contributing to the development of metabolic dysfunction-associated steatotic liver disease, such as nonalcoholic fatty liver disease (NAFLD) (28). In our previous study, *Morc4* has been identified as a central hub gene within the network of lipid metabolism co-expression genes (13). This study elucidates the significant correlation between *Morc4* expression and lipid metabolism, utilizing BXD recombinant inbred strains and the IMPC database, and further explores the associated mechanisms through *in vitro* experiments.

A systematic genetic analysis reveals a significant negative correlation between hepatic *Morc4* expression and the levels of plasma free fatty acids and triglycerides. *in vitro* experiments, *MORC4 g*ene silencing elevated TC and TG levels, with lipid droplet accumulation observed in both normal and OA-treated cells, whereas *MORC4* overexpression restored total TC and TG levels, along with lipid accumulation in these cell models. Similar phenotypes are also observed in IMPC database. Compared with wild-type mice, *Morc4* knockout mice exhibit elevated total cholesterol levels and cholesterol ratios, as well as an increase in fat mass and fat/body weight ratios. Collectively, these findings underscore the critical role of MORC4 in maintaining hepatic lipid metabolism homeostasis. To further investigate the potential influence of MORC4 on lipid metabolism signaling pathways, 2,000 genes that were co-expressed with *Morc4* expression in the liver were identified and analyzed. Notably, many of these genes are involved in cholesterol metabolism including *Pcsk9*, *Lipa*, *Lipg*, *Pltp*, *Apoc2*, *Cd36*, and *Apoa4*.

Our findings reveal a significant negative correlation between *Morc4* and *Pcsk9* expression, while a significant positive correlation exists between *Morc4* expression and that of *Pltp* and *Cd36*. PCSK9 is a hepatic protease that facilitates the degradation of low-density lipoprotein receptors (LDLRs) (29, 30). LDLRs are responsible for the uptake of LDLs from the bloodstream, subsequently transporting them to the liver for degradation, which releases cholesterol (31). Gain-of-function mutations in *Pcsk9* are linked to familial hypercholesterolemia and the inhibition of PCSK9 is emerging as a novel strategy for the treatment of hypercholesterolemia (32, 33). PLTP plays a crucial role in the transport of cholesterol to the liver for its degradation and excretion (34, 35). CD36 functions as a fatty acid transporter, contributing to the cellular uptake of fatty acids (36, 37). A reduction or loss of function in both PLTP and CD36 may lead to lipid accumulation and metabolic dysfunction (36, 38). *MORC4* knockdown results in an upregulation of *PCSK9* level and a downregulation of *PLTP* and *CD36* levels. Furthermore, *MORC4* overexpression restored the expression changes of these genes induced by MORC4 knockdown. These results suggest that MORC4 may play a regulatory role in lipid metabolism by influencing the uptake of free fatty acids and cholesterol.

Our research indicates a significant positive correlation between *Morc4* expression and the levels of *Apoc2*, *Apoa4*, *Lipg* and *Lipa,* all of which play essential roles in triglyceride metabolism. APOC2 could activates lipoprotein lipase for the hydrolysis of hydrolysis of triglyceride-rich plasma lipoproteins, a process that is critically supported by APOA4 (39, 40). *Apoc2* deficiency elicits severe hypertriglyceridemia (41). Additionally, LIPG and LIPA are involved in the hydrolysis of triglycerides (42, 43). Moreover, our findings imply a significant negative correlation between *Morc4 and Hmgcr* expression. HMGCR serves as a key rate-limiting enzyme in *de novo* cholesterol synthesis (44, 45). An increase in *HMGCR* expression enhances cholesterol synthesis, while its inhibition leads to a reduction in serum cholesterol levels (46, 47). *in vitro* experiments, *MORC4* knockdown is associated with a reduction in the expression of *APOC2*, *APOA4*, *LIPG* and *LIPA*, alongside an increase in *HMGCR* expression. Whereas, MORC4 overexpression in cells with MORC4 knockdown reinstates the expression levels of these genes. These results suggest the critical role of MORC4 in the regulation of cholesterol synthesis and metabolism.

Research on the regulatory mechanisms of MORC4 remains limited. A Study in HEK293 T cells shows that MORC4 mediates nuclear body formation and inhibits binding of transcription factors (48). In Arabidopsis, MORC4 was implicated in DNA methylation establishment (49). These findings suggest MORC4 may suppress *PCSK9* and *HMGCR* expression by enhancing promoter methylation. However, the mechanisms through which MORC4 upregulates genes such as *LIPA* still remain to be investigated. Furthermore, targeting *PCSK9* to enhance its methylation has been proposed as a therapeutic strategy for hypercholesterolemia (50). Consequently, MORC4 may also represent as a potential therapeutic target for the treatment of hypercholesterolemia.

In addition, an elevated expression of *PCSK9, LIPA*, *LIPG*, *PLTP* and *APCO2* was noted in OA-treated THLE-2 and HepG2 cells. Conversely, *Hmgcr* expression was found to be downregulated in these OA-treated cells. These findings suggest that cholesterol synthesis is inhibited while cholesterol uptake and triglyceride hydrolysis are activated, thereby contributing to the maintenance of intrahepatic lipid homeostasis in hyperlipidemic milieu. The expression of *MORC4* was also increased in OA-treated cells. It aligns with its role in negatively regulating cellular triglyceride and cholesterol levels, as indicated by our results. However, the expression of *PLTP* and *CD36* was downregulated in OA-treated cells, potentially due to their primary function in lipid absorption within the vascular compartment (51, 52). Notably, the levels of PLTP have been reported to be elevated in the liver of mice fed a high-fat high-cholesterol (HFHC) diet, as well as in the plasma of patients with NAFLD (53, 54).

In summary, utilizing the BXD genetic reference population, alongside integrative approaches from systems genetics and molecular biology, we identified MORC4 as a critical determinant of hepatic lipid metabolism. MORC4 exerts its influence on lipid metabolism via three distinct pathways (Figure 5G). First, MORC4 influences hepatic uptake of free fatty acids and cholesterol from plasma through PCSK9, CD36 and PLTP. Second, it regulates the hydrolysis of triglycerides through APOC2, APOA4, LIPG, and LIPA. Lastly, Morc4 plays a role in the modulation of cholesterol synthesis through HMGCR. Our research reveals the regulator function of MOCR4 in lipid metabolism homeostasis. Dysregulation of liver metabolism can lead to liver diseases such as fatty liver and hepatitis, hepatocyte damage and abnormal liver function. It may also induce metabolic syndromes like obesity, diabetes, and hyperlipidemia, increasing the risk of cardiovascular diseases. Consequently, MORC4 may represent a promising therapeutic target for the treatment of metabolic syndromes. Take together, our results enhance understanding of the regulatory networks governing lipid metabolism but also offer novel perspectives for the personalized treatment of metabolic diseases.

## Data Availability

The original contributions presented in the study are included in the article/Supplementary Material, further inquiries can be directed to the corresponding author.
