# Supplementary material for: Morc4 is a novel functional gene associated with lipid metabolism in BXD recombinant inbred population
Source: Front Cardiovasc Med. 2025 Jun 18;12:1570729. doi: 10.3389/fcvm.2025.1570729 (PMC12217220; doi:10.3389/fcvm.2025.1570729)
Supplement: Supplementary file 2 [file Image1.pdf]

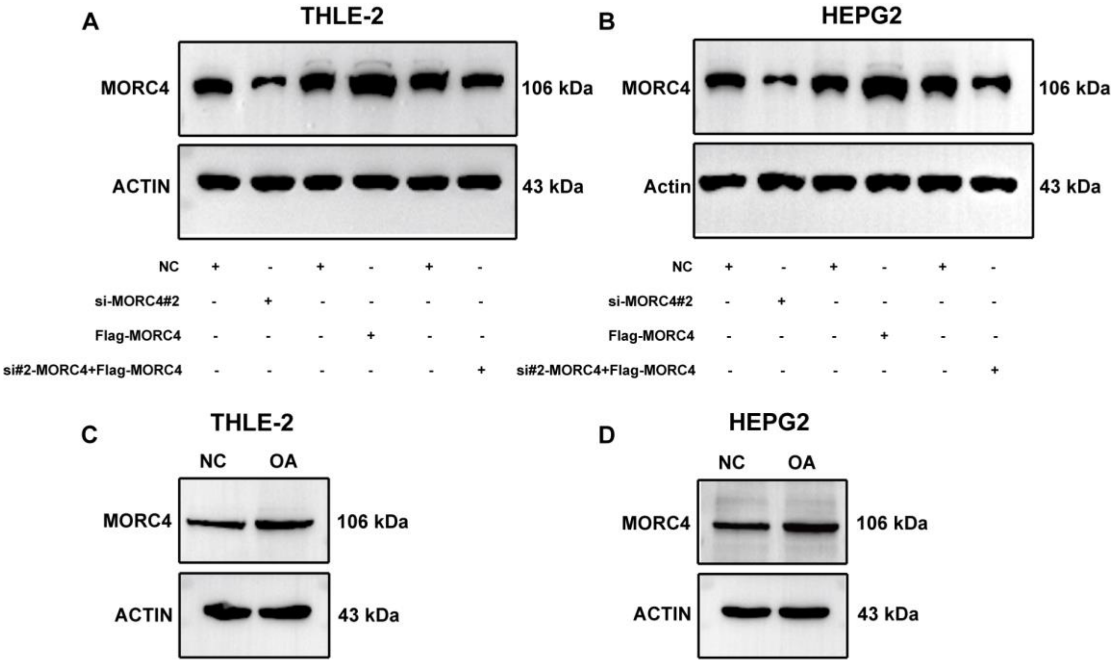

Supplementary Figure 1. MORC4 protein expression was verified by West Blot analysis. The knockdown and overexpression of MORC4 in THLE-2 (A) and HepG2 (B) cells were confirmed with West Blot analysis. The MORC4 protein expression in THLE-2 (C) and HepG2 (D) cells after OA-treatment were examined with West Blot analysis.
